# Long-term adaptive response in COVID-19 vaccine recipients and the effect of a booster dose

**DOI:** 10.3389/fimmu.2023.1123158

**Published:** 2023-02-28

**Authors:** Luca Perico, Marta Todeschini, Federica Casiraghi, Marilena Mister, Anna Pezzotta, Tobia Peracchi, Susanna Tomasoni, Piera Trionfini, Ariela Benigni, Giuseppe Remuzzi

**Affiliations:** Department of Molecular Medicine, Istituto di Ricerche Farmacologiche Mario Negri IRCCS, Bergamo, Italy

**Keywords:** COVID-19, SARS-CoV-2, Delta, Omicron, mRNA vaccine, neutralizing antibodies, T and B cells

## Abstract

We examined the immune response in subjects previously infected with SARS-CoV2 and infection-naïve 9 months after primary 2-dose COVID-19 mRNA vaccination and 3 months after the booster dose in a longitudinal cohort of healthcare workers. Nine months after primary vaccination, previously infected subjects exhibited higher residual antibody levels, with significant neutralizing activity against distinct variants compared to infection-naïve subjects. The higher humoral response was associated with higher levels of receptor binding domain (RBD)-specific IgG^+^ and IgA^+^ memory B cells. The booster dose increased neither neutralizing activity, nor the B and T cell frequencies. Conversely, infection-naïve subjects needed the booster to achieve comparable levels of neutralizing antibodies as those found in previously infected subjects after primary vaccination. The neutralizing titer correlated with anti-RBD IFNγ producing T cells, in the face of sustained B cell response. Notably, pre-pandemic samples showed high Omicron cross-reactivity. These data show the importance of the booster dose in reinforcing immunological memory and increasing circulating antibodies in infection-naïve subjects.

## Introduction

As of February 2023, the coronavirus disease 2019 (COVID-19) pandemic had resulted in over 670 million severe acute respiratory syndrome coronavirus 2 (SARS-CoV-2) infections and almost 6.8 million deaths worldwide (1). One key step in limiting the spread of SARS-CoV-2 and severe clinical outcomes in COVID-19 has been the development of effective and durable vaccine protection. Two mRNA vaccines that exhibited strong immunogenicity and efficacy were approved (2, 3) between December 2020 and February 2021.

Despite initial encouraging results in clinical trials, an increase in breakthrough SARS-CoV-2 infections over time in vaccinated individuals has raised concerns about the long-term efficacy of these vaccines in the real world, as well as their efficacy against new, emerging variants (4). Most of the available clinical and modeling studies suggested that increased breakthrough infections could be the result of a reduction in circulating antibody levels between 4 and 6 months after primary vaccination (5–8).

However, several groups have consistently documented that functional preservation of T cell responses following primary vaccination (9–12) could play an important role as a second-level defense against SARS-CoV-2. These results could explain the finding that a putative reduction in vaccine efficacy against infection with a SARS-CoV-2 variant did not result in a parallel decline in protection against severe disease, which was still apparent up to 9 months after primary vaccination (13, 14).

In mid-2021, the rapid emergence and spread of various SARS-CoV-2 variants with high infectivity and transmissibility, such as the Delta variant, which may elude vaccine-induced humoral immunity (15), prompted some countries to offer an additional booster dose to those who have received a primary vaccination (16). Despite the uncertainty, the booster dose was recommended to subjects at higher risk of developing severe COVID-19, such as the elderly and immunocompromised subjects, as well as to subjects at high risk of infection, such as healthcare workers (HCWs). The booster dose was recommended 4 to 6 months after the primary vaccination, to address potentially decreasing humoral immunity and to restore vaccine efficacy against infection with different emerging variants (17). Therefore, all of the studies that are currently available have investigated the immunological response to primary vaccination for up to 6 months, while the long-term response beyond this time point remained largely unexplored.

Here, using a cohort of healthcare workers (HCWs), we investigated the humoral and cellular response 9 months after primary BNT162b2 (Comirnaty, Pfizer-BioNTech) vaccination, with a special focus on the variants that have emerged most recently, Delta and Omicron. We also evaluated longitudinal immunological changes over a 3-month period following homologous booster dose administration in the same vaccine recipients.

## Methods

### Ethics statement

The ADAPTIVE study, involving human subjects, was reviewed and approved by the Ethical Committee of the Istituto Nazionale per le Malattie Infettive Lazzaro Spallanzani IRCCS (PARERE N. 444_2021). The study conforms to the principles of the Helsinki Declaration and written informed consent was obtained from all enrolled subjects. Study participation was voluntary. No potentially identifiable human images or data are presented in this study.

### Enzyme-linked immunosorbent assay

Human IgG against the RBD of the spike protein of SARS-CoV-2 were measured using a quantitative ELISA (Proteintech, #KE30003). Briefly, serum samples diluted 1:200 were incubated on 96-microwell plates pre-coated with recombinant S-RBD recombinant protein. Captured anti-S-RBD human antibodies were detected using HRP-conjugated secondary antibodies against anti-human IgG. Averages of duplicate readings for each standard and sample were subtracted for the average zero standard absorbance. Data were obtained with a best-fit standard curve determined by regression analysis using four-parameter logistic curve fit (4-PL) and expressed as μg/mL. The threshold for sample positivity for anti-S-RBD antibodies was set by the manufacturer as > 0.625 μg/mL. To monitor SARS-CoV-2 infection during the study period, a quantitative ELISA was used to detect IgG against the SARS-CoV-2 nucleocapsid protein (Proteintech, #KE30001).

### Cell culture and lentiviral neutralization assay

Vero E6 cells (ATCC, C1008; RRID: CVCL_0574) were cultured in Minimum Essential Medium Eagle EBSS with L-Glutamine (EMEM, Lonza, #BE12611F) supplemented with 1% non-essential amino acids, 10% heat inactivated fetal bovine serum (FBS; Life Technologies, #10270106) and 1% penicillin/streptomycin (P/S; Life Technologies, #15140122).

To potentiate lentivirus infection, we over-expressed in Vero E6 cells the main receptor involved in SARS-CoV-2 infection, angiotensin converting enzyme 2 (ACE2). Briefly, parental cells were transfected with replication incompetent, HIV-based, VSV-G pseudotyped ACE2 lentivirus (BPS bioscience, #79944). Specifically, 500,000 cells/well (6-well culture plate) were transduced with 10 M.O.I *per* cell of ACE2 lentivirus in the presence of 5 μg/mL of polybrene (Sigma Aldrich, #TR-1003). After 52 hours of transduction, the ACE2 overexpressing Vero E6 cells were harvested and seeded, 5,000 cells per well (96-well culture plate). The following day, cells were exposed to 2 M.O.I. *per* cell of an enhanced green fluorescent protein (eGFP) pseudotyped lentivirus expressing the spike protein of SARS-CoV-2 B.1.617.2 (Delta; BPS bioscience, #78216) or the spike protein of SARS-CoV-2 B.1.1.529.1 (Omicron; BPS bioscience, #78349) in the presence of 5 μg/mL polybrene overnight at 37°C, 5% CO_2_. A bald lentiviral pseudovirion with eGFP reporter (BPS bioscience, #79987) was used at the same concentration as a negative control to confirm the spike-dependent pseudovirus infection.

To test the sera neutralizing activity, pseudotyped lentivirus were pre-incubated for 30 minutes with randomly selected sera from vaccinated individuals (1:200 dilution) before incubation with ACE2 overexpressing Vero E6 cells. After 24-hour incubation, infection medium was discarded and 500 µL of fresh EMEM medium was added to each well. After 48 hours, cells were fixed and monitored under ApoTome Axio Imager Z2 (Carl Zeiss) to assess eGFP positivity. Before fixation nuclei were counterstained with Hoechst (NucBlue^®^ Live ReadyProbes^®^; Thermo Fisher, Invitrogen, #R37605). At least 15 field *per* sample were acquired and the number of eGFP-positive Vero E6 cells counted (cells/field). The neutralizing activity was assessed as the ability of sera to reduce the number of infected cells and expressed as the percentage of reduction (%) in eGFP-positive cells exposed to lentiviral constructs pre-exposed to sera compared to the eGFP-positive cells exposed to lentiviral constructs alone.

### B cell analysis

Peripheral blood mononuclear cells (PBMC) were isolated by gradient density centrifugation (Ficoll-Paque Plus, GE Healthcare, #17-1440-03). Frozen PBMC were thawed in complete RPMI medium (Thermo Fisher, #61870036) plus 5% human serum AB (Euroclone, #ECS0219D). B cells (8-10 million/each protein) specific for spike protein (Miltenyi, #130-1289-022), RBD protein (Miltenyi, #130-128-032) and for the spike B.1.1.529.1 – Omicron variant (Acro Biosystems, #SPN-C82Ee) were evaluated by double tetramer staining using specific B cell analysis kits (Miltenyi, #130-128-032 and #130-128-022), following the instructions (8-10 million PBMC/each protein). Data were acquired on FACS LSRFortessa X-20 (BD Biosciences, San Jose, CA, USA) and analyzed with FlowJo software (FlowJo LLC). Live singlets were gated based on 7AAD fluorescence and specific B cells detected using the double discrimination method gated on CD3^-^ CD14^-^ 7AAD^-^ and CD19^+^ cells (CD3 PerCP-Vio700, #130-113-132; CD14 PerCP-Vio700, #130-113-151, Miltenyi). Cells incubated with streptavidin PE and PEVio770 alone were used as negative controls. Specific memory B cells were defined as CD27^+^ CD19^+^ on tetramer^+^ B cells.

### IFNγ enzyme-linked immunosorbent spot assay

PBMC (1.5x10^6^/mL) were stimulated with 1 µg/mL of peptide pools covering complete SARS-CoV-2 spike protein (PepTivator SARS-CoV-2 Prot-S Complete, Miltenyi, #130-127-953), the receptor binding domain RBD 319-541 (JPT, #PM-WCPV-S-RBD-2), the spike protein of the BA.1 Omicron variant (SARS-CoV-2 Prot-S B.1.1.529/BA.1 Mutation Pool, Miltenyi, #130-129-928) in an IFNγ ELISPOT assay (TEMA Ricerca, #856.051.010). Cells incubated with dimethyl sulfoxide (DMSO, Sigma, #D2438) were used as negative controls, while cells stimulated with CEFX Ultra SuperStim pool (JPT, #PM-CEFX-1) were used as positive stimulation controls. Cells (300,000 PBMC/well) were stimulated for 20 hours at 37°C 5% CO_2_ in three replicates and then the ELISPOT assay was carried out according to manufacturer’s instructions. To quantify peptide-specific response, spots of the DMSO (usually less than 20) were subtracted from the peptide stimulation wells and the results expressed as spots/300,000 PBMC.

Compared to the activation-induced marker (AIM) assays (11, 12), in our hands the IFNγ ELISPOT assay provided more solid and wider differences between positive and negative controls and peptide stimulation wells. The negative control (DMSO) produced a very low number of spots while the spots in the positive control (CEFX) were consistently high in all subjects (over 200 spots/300,000 PBMC).

### Statistical analysis

Data were reported as mean ± standard deviation or number (%). Differences between groups were evaluated by unpaired t-test or Fisher’s exact test as appropriate. Continuous levels of cellular response against SARS-CoV-2 were expressed as median [interquartile range] and group comparisons were performed by non-parametric test Wilcoxon rank sum test. Correlations between continuous variables were evaluated with Pearson’s index. Data were presented as box-and-whisker plots displaying the median, 25th and 75th percentiles of distribution and whiskers extend to the minimum and maximum values of the series. All analyses were carried out using SAS (version 9.4). All *p-values* were 2-sided.

For the analysis of the lentiviral infection assay, data were expressed as mean ± standard deviation and comparisons were made using ANOVA with corrected with Tukey *post hoc* test.

## Results

**Figure 1:**
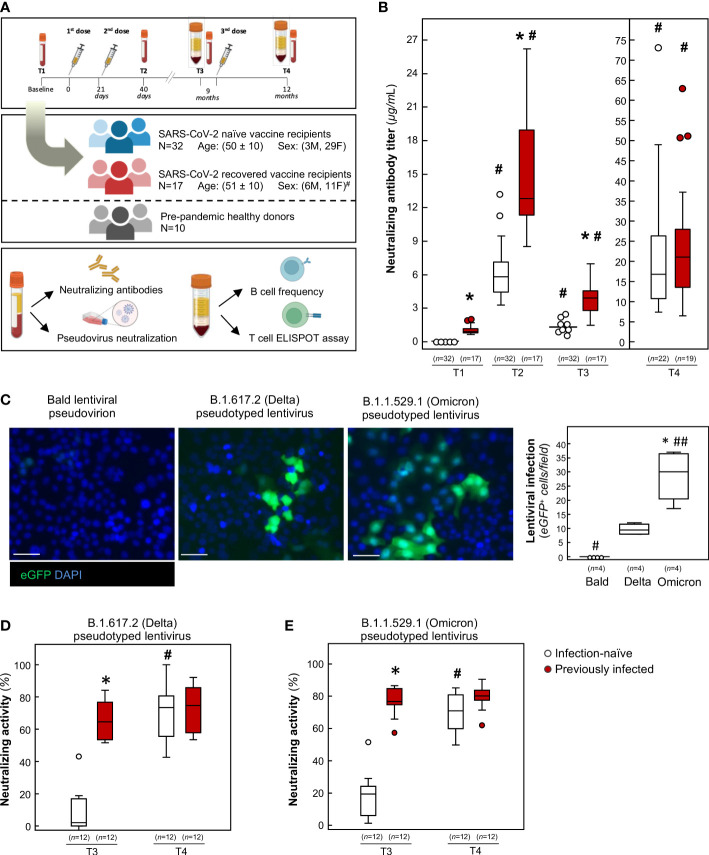
Humoral response and neutralizing activity of infection-naïve and previously infected vaccine recipients over time. **(A)** Schematic representation depicting the study design. Drawings were created using BioRender. #*p-value*=0.049 *vs* infection-naïve subjects. **(B)** Evaluation of neutralizing anti-RBD IgG in infection-naïve and previously infected vaccine recipients at baseline (T1), 19 days after primary vaccination (T2), 9 months after primary vaccination (T3), and 3 months after the booster dose (T4). *p-value<0.0001 *vs* infection-naïve subjects; #*p-value*<0.0001 *vs* the respective T1. **(C)** Representative images and quantification of lentiviral construct infection in Vero E6 cells overexpressing human angiotensin converting enzyme 2 (ACE2). Nuclei are counterstained with Hoechst. Scale bar: 50 μm. *p-value<0.001 *vs* Bald, #*p-value*<0.01; ##*p-value*<0.001 *vs* Delta. **(D, E)** Quantification of neutralizing activity of sera against **(D)** B.1.617.2 (Delta) and **(E)** B.1.1.529.1 (Omicron) at T3 and T4. *p-value<0.0001 *vs* infection-naïve subjects; #*p-value*<0.0001 *vs* the respective T3. The sample size (*n*) for each panel is indicated in brackets.

This observational study included a total of 49 HCWs from the Mario Negri Institute’s Clinical Research Centre. Baseline characteristics are reported in **Figure 1A**. On January 10, 2021, all HCWs received a primary vaccination with BNT162b2, according to the standard regimen of 2 doses administered 3 weeks apart. A baseline blood withdrawal was performed before vaccination (T1) and a subsequent withdrawal was scheduled 19 days after the second dose (T2) to obtain serum samples for antibody evaluation.

To evaluate the levels of neutralizing antibodies, we used an enzyme-linked immunosorbent assay (ELISA) pre-coated with recombinant receptor binding domain (RBD), the main region of the SARS-CoV-2 spike (S) protein involved in viral entrance into target cells (18, 19). As shown in **Figure 1B**, baseline evaluation (T1) identified anti-RBD antibody levels above the detection threshold in 17 HCWs (35%), who were categorized as previously infected. These subjects encountered SARS-CoV-2 synchronously during the peak of the first wave of the pandemic caused by Wuhan Hu-1 in March 2020 in northern Italy (20). When we analyzed the anamnestic questionnaires completed by previously infected volunteers, we found that COVID-19 mostly presented as a mild disease, with fever, muscle pain and fatigue the most commonly experienced symptoms and none of the infected subjects requiring hospitalization (**Table S1**). On the other hand, 32 HCWs (65%) tested negative for anti-S-RBD antibodies and were considered naïve to natural infection (**Figure 1B**). Mean antibody levels were 1.05 ± 0.44 and 0.08 ± 0.03 μg/mL in previously infected and infection-naïve individuals, respectively, at T1 (**Figure 1B**). Nineteen days after the primary vaccination (T2), all HCWs had mounted a robust neutralizing humoral response, although vaccine-evoked humoral response was significantly higher in previously infected subjects (**Figure 1B** **)**. In line with other studies (21–27), our data indicate that response to primary vaccination is associated with a greater neutralizing antibody titer in individuals with a previous history of SARS-CoV-2 infection.

Following the approval of the booster dose by European regulatory agencies, all subjects received the BNT162b2 booster in November 2021. Before the booster dose, all available consenting HCWs underwent a blood withdrawal (T3) to obtain sera for antibody evaluation and peripheral blood mononuclear cells (PBMC) for T and B cell analysis. During the period between the primary vaccination and blood withdrawal before the booster dose (T3), no SARS-CoV-2 infections were reported in infection-naïve HCWs when they were tested using real-time reverse transcription polymerase chain reaction (28) or ELISA for anti-nucleocapsid antibodies. A blood sampling repeat was planned on February 2022, 3 months after the booster dose (T4). During the period between the booster dose and the blood withdrawal at T4, 3 infection-naïve HCWs were diagnosed with SARS-COV-2 using qRT-PCR tests and tested positive for anti-nucleocapsid antibodies. These 3 individuals were moved to the previously infected group in the analysis at T4.

Analysis of anti-S-RBD antibody titer at 9 months (T3) revealed that neutralizing antibodies decreased substantially and to a similar extent in both groups (**Figure 1B**, decrease: 73.9 ± 9.9% *vs* 75.9 ± 7.0%, mean ± standard deviation), demonstrating that previous SARS-CoV-2 infection did not alter rates of antibody reduction over time. Despite this marked decline, both groups were still positive for anti-S-RBD IgG, and none of the subjects had dropped to subthreshold levels (**Figure 1B**). Our data extend the observations made by Goel and colleagues, who reported detectable neutralizing antibodies in most vaccine recipients for up to 6 months following primary vaccination (29, 30), even in the setting of a different mRNA vaccine (31). At T4, we found that the booster dose significantly restored neutralizing antibody levels, to a similar extent in both groups (**Figure 1B**).

When the study was completed (1 May 2022), 6 additional SARS-CoV-2 diagnoses were reported in the infection-naïve group after blood withdrawal at T4, and 2 were reported in previously infected subjects. Altogether, a total of 9 naïve subjects (28.1%) experienced SARS-CoV-2 infection after the booster dose, while only 2 infections (11.8%) were reported in previously infected subjects.

In order to assess whether the residual antibody levels detected at 9 months had a neutralizing effect on the most recent SARS-CoV-2 variants, we performed a lentiviral infection assay with an enhanced green fluorescent protein (eGFP) pseudotyped lentivirus that expressed the full length spike protein of either the B.1.617.2 (Delta) or B.1.1.529.1 (Omicron) lineages. As shown in **Figure 1C**, exposing the cells to the Delta lentivirus was associated with a lower frequency of eGFP expression compared to Omicron. No signal was observed when a bald lentiviral pseudovirion was used as a negative control (**Figure 1C**), confirming the spike-dependent lentiviral infection of target cells. In this setting, we tested the antibody-neutralizing activity by incubating lentiviral constructs with serum samples. As shown in **Figure 1D**, the residual antibody levels in previously infected HCWs retained potent neutralizing capacity at 9 months, as demonstrated by the ability of sera to halt the Delta pseudotyped lentivirus infection in target cells. Similarly, residual neutralizing antibody blocked Omicron lentiviral infection to a significant extent (**Figure 1E**). Our data extend the observations made by Luczkowiak and colleagues to Omicron. They had reported that COVID-19 patients who had recovered had strong neutralizing antibody titers against previous variants of concerns 8 months after primary vaccination (32). Given that several studies have reported that residual neutralization levels may still be sufficient to protect against symptomatic disease (33–35), our finding supports the hypothesis that previously infected vaccine recipients are protected at 9 months. In line with this hypothesis, a recent study documented that infection-acquired immunity, in combination with primary vaccination, conferred a high level of protection against SARS-CoV-2 more than 1 year after infection (36), even against the BA.5 variant that had recently emerged (37). This finding was also observed in high-risk populations (38). Conversely, sera from infection-naïve subjects collected 9 months after primary vaccination exhibited no neutralizing activity toward either the Delta or Omicron constructs (**Figure 1D, E**). Our findings are in line with data that show suboptimal post-vaccine immune responses in infection-naïve individuals (39, 40).

In previously infected subjects, the upsurge of neutralizing antibody levels following the booster dose was not paralleled by a comparable increase in neutralizing activity, which was only slightly enhanced (**Figures 1D, E**). In contrast, the vigorous upsurge in neutralizing antibody titer in infection-naïve individuals was associated with a significant increase in neutralizing activity against different variants (**Figures 1D, E**), suggesting that these subjects require a booster dose to achieve appropriate neutralizing activity against both SARS-CoV-2 variants (41). Our results are fully consistent with those from a different cohort which showed that, almost 9 months after primary vaccination, Delta neutralization was detected in only 19% of COVID-19 naïve subjects and 88% of subjects who had recovered from COVID-19 (42). As in our study, a booster dose was required to restore neutralizing activity against Delta in COVID-19 naïve vaccine recipients. These data are confirmed by real word data from two independent studies that show that vaccine efficacy against infection with the Delta variant was around 80% soon after primary vaccination (<120 days), while it decreased over time to 0-50% (>120 days) (43, 44). In both studies, vaccine efficacy against infection with the Delta variant was restored by the booster dose. Collectively, these data may suggest that immune protection against infection needs to be optimized through the booster dose for infection-naïve subjects.

**Figure 2:**
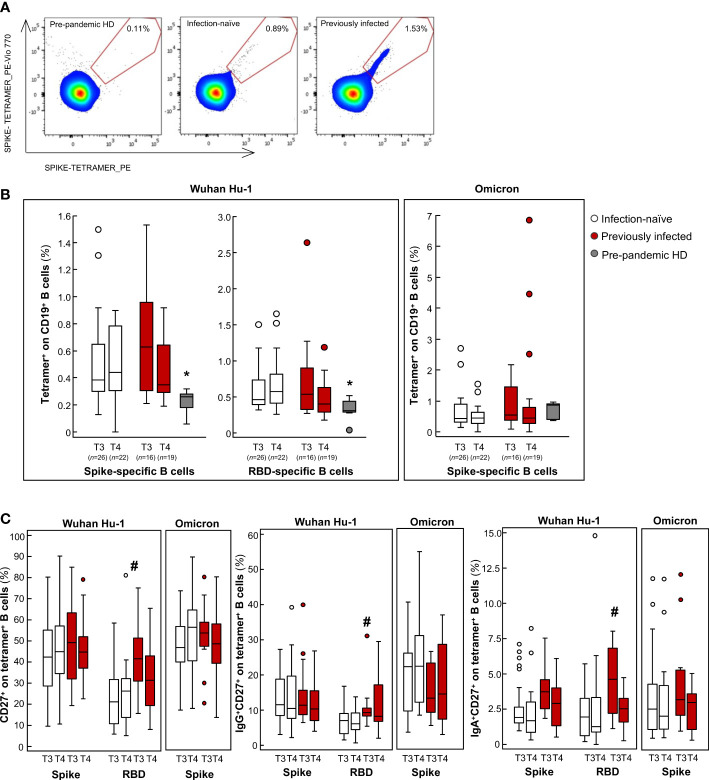
Analysis of B cell frequency in infection-naïve and previously infected vaccine recipients over time. **(A)** Representative flow cytometry pseudocolor plots of spike-specific B cells in pre-pandemic healthy donor (HD), in an infection-naïve and a previously infected subject. **(B, C)** Percentages of tetramer^+^ on CD19^+^ B cells **(B)** and the percentages of memory CD27^+^, IgG^+^CD27^+^ and IgA^+^CD27^+^ B cells on tetramer^+^ B cells **(C)** are shown for the spike and RBD protein of the Wuhan Hu-1 SARS-CoV-2 and in response to the B.1.1.529.1 (Omicron) spike protein in infection-naïve and previously infected subjects at 9 months after primary vaccination (T3) and 3 months after the booster dose (T4), as well as for pre-pandemic HD. *p-value<0.05 *vs* infection-naïve and previously infected subjects at T3 and T4; #*p-value*<0.05 *vs* infection-naïve subjects at T3. The sample size (*n*) for all B cell analyses is indicated in brackets in panel **(B)**.

We next investigated the B cell response to mRNA vaccination. Indeed, previously published studies have shown that mRNA vaccines generate functional memory B cells and that levels of these cells increase 3 months after primary vaccination (29) and persist until 6 months post-primary vaccination, despite the marked decrease in specific IgG neutralizing antibodies (45–48). Based on these findings, we sought to evaluate whether changes in SARS-CoV-2-specific B cell frequencies were responsible for the changes observed in the neutralizing activity. To this end, we used a double tetramer fluorescence activated cell sorting (FACS) staining approach to quantify memory B cells specific to the spike protein and the RBD of Wuhan Hu-1. To evaluate the magnitude of the specific B cells that recognize SARS-CoV-2, we also analyzed PBMCs from healthy donors, which had been collected before 2019. Representative flow cytometry pseudocolor plots of spike-specific B cells are shown in **Figure 2A**. Compared to pre-pandemic healthy donors, in vaccinated individuals at T3 and T4, circulating B cells specific for Wuhan Hu-1 spike and RBD were significantly higher (**Figure 2B**). No major differences were observed in the B cell frequency between previously infected and infection-naïve subjects (**Figure 2B**). When we further analyzed the specific CD27^+^ memory B cell subsets, we found that Wuhan Hu-1 RBD-specific CD27^+^ memory B cells and, in particular IgG^+^CD27^+^ and IgA^+^CD27^+^, but not IgM^+^CD27^+^, were significantly higher at 9 months in previously infected subjects compared to infection-naïve subjects (**Figure 2C**). Notably, anti-S-RBD IgG^+^CD27^+^ and IgA^+^CD27^+^ B cells significantly correlated with neutralizing antibody at T3 (**Table S2**). Although we did not investigate anti-S-RBD IgA levels, the finding that IgA^+^CD27^+^ RBD-specific memory B cells correlated positively with neutralizing antibody suggests that previously infected vaccine recipients have additional neutralizing protection, as IgA has been shown to mediate the early SARS-CoV-2 neutralizing response (49). This hypothesis has been confirmed by recent data that showed that vaccination induced a minimal IgA response in individuals who had not been exposed to SARS-CoV-2, while IgA induction after vaccination was more efficient in patients with a COVID-19 history (50). A recent study also documented the critical role that IgA^+^ B cell memory recall induced by vaccination plays in breakthrough infection (51). These data provide a novel insight into long-term immunity to SARS-CoV-2 in previously infected subjects who exhibited marked immunological imprinting from previous infection, shaping the long-term breadth and maturation of neutralizing activity, even in the absence of a booster dose. These data indicate that pre-vaccination immunological memory plays a major role in dictating better vaccination outcomes and may explain why fewer breakthrough infections were reported in our cohort of previously infected subjects, in line with real-world data (52). After the booster dose, we did not find any changes in either the frequency of B cells specific for the spike protein of Wuhan Hu-1 (**Figure 2B**) or in the number of IgG^+^ and IgA^+^ CD27^+^ memory B cells (**Figure 2C**). These data indicate that the booster dose does not induce a major expansion of memory B cells, either in previously infected or in infection-naïve subjects. These findings apparently contrast with those reported recently by Goel and colleagues, who documented a significant expansion of the memory B cell repertoire following the booster dose (53). However, in their analysis the authors showed that the greatest expansion of spike-specific memory B cell was detected at 2 weeks post-booster, while it declined over the following months (53). Having investigated the B cell response 3 months after the booster dose, it is conceivable that we missed the early transient expansion of spike-specific B cells triggered by the antigenic stimulus of the booster dose. All these data suggest that, regardless of the rapid and transient immunogenic stimulus provided by the booster dose, the B cell magnitude is durable and effective in producing a large amount of neutralizing antibodies when faced with additional antigen exposure (51).

**Figure 3:**
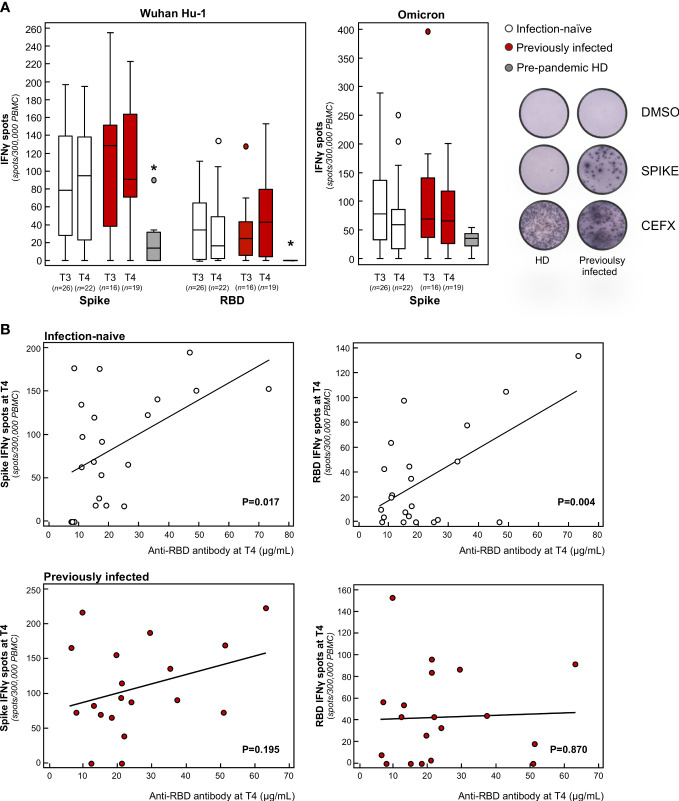
Analysis of effector T cell response in infection-naïve and previously infected vaccine recipients over time. **(A)** Frequency of IFNγ producing T cells in response to peptide pools of the spike and RBD protein of the Wuhan Hu-1 SARS-CoV-2 and in response to the B.1.1.529.1 (Omicron) spike protein in infection-naïve and previously infected subjects at 9 months after primary vaccination (T3) and 3 months after the booster dose (T4), as well as in pre-pandemic healthy donors (HD). Representative ELISPOT wells are shown on the right. Horizontal lines indicate median values; *p-value<0.005 *vs* of infection-naïve and previously infected subjects at T3 and T4. **(B)** Correlation of anti-RBD antibody levels with the frequency of spike-specific (left panels) or RBD-specific (right panels) IFNγ producing T cells at T4 in the two study groups. The sample size (*n*) for all T cell analyses is indicated in brackets in panel **(A)**.

To analyze the full spectrum of cellular immunity, we focused on T cells, given that their response to the spike protein is instrumental in the coordinated humoral response that follows primary mRNA vaccination (54). To assess the total effector T cell response, we performed an IFNγ ELISpot assay following stimulation with pooled overlapping 15-mer peptides spanning the full length and RBD of Wuhan Hu-1. In this setting, the specific IFNγ T cell responses against the spike and RBD were significantly higher in all vaccine recipients at 9 months (T3) compared to pre-pandemic healthy donors, with no difference between previously infected and infection-naïve subjects (**Figure 3A**). After the booster dose, no major differences were found between the vaccinated groups in terms of the magnitude of spike-specific T cell responses (**Figure 3A**). In a recent study, Naranbhai and colleagues reported that T cell reactivity to the SARS-CoV-2 spike protein was enhanced significantly after the booster dose, particularly in previously infected subjects (55). However, these data were obtained from samples collected soon after the booster dose, reflecting a transient response to the antigenic stimulus (55). The short-term nature of the acute T cell response following the booster dose was confirmed across different age groups (56–58).

In our study, we also found that the effector spike- and RBD-specific T cell response correlated significantly with neutralizing antibody titer in infection-naïve but not in previously infected subjects (**Figure 3B**). Our results are in line with three independent studies that showed that the SARS-CoV-2-specific T cell response is required to induce long-term persistence of neutralizing antibodies during natural infection (59, 60), as well as in response to primary vaccination (61). On top of that, our study indicates that the additional antigenic challenge – through the booster dose – is essential for infection-naïve individuals to mount a coordinated T cell response, which sustains the neutralization breadth against SARS-CoV-2 variants. This finding is of particular clinical relevance considering the transient increase stimulation of T cells following the booster dose has been associated with enhanced affinity maturation of RBD-specific IgG in a cohort of adults above the age of 80 who were at risk of severe disease (56).

When we analyzed the B and T cell response against Omicron, we found that, after primary vaccination, subjects exhibited a B cell frequency and an IFNγ T cell response to the spike protein of Omicron that was comparable to the response to Wuhan Hu-1, with no additional changes following the booster dose (**Figures 2B**, **3A**). Notably, the cellular responses against Omicron, but not Wuhan Hu-1, were also observed in pre-pandemic samples from healthy donors (**Figures 2B**, **3A**). There was no difference in the extent of B and T cell responses between vaccinated individuals and healthy donors (**Figures 2B**, **3A**). Our data are consistent with the presence of cross-reactive B cells against the non-RBD portions of Omicron in unvaccinated uninfected individuals (62) with no pre-existing B cell immunity against the spike protein of SARS-CoV-2 of the Wuhan Hu-1 variant (63). As for memory B cells, there were no differences in terms of either the frequency or phenotype of CD27^+^ B cells between previously infected and infection-naïve subjects (**Figure 2C**). Omicron RBD-specific IgG^+^CD27^+^ B cell response positively correlated with neutralizing antibody levels in previously infected subjects (**Table S2**), which may explain why serum samples from these individuals strongly neutralized Omicron lentiviral infection 9 months after primary vaccination. Regarding T cells, to the best of our knowledge only two studies have investigated the response of IFNγ T cells to Omicron in pre-pandemic samples using the ELISPOT assay. In a pre-print study, Jergovic and colleagues found that T cell cross-reactivity to the spike protein of Wuhan Hu-1 was slightly higher than that of Omicron in samples from healthy adults collected prior the pandemic (64). Conversely, Naranbhai and colleagues found that the T cell response against the spike protein was low in 10 non-vaccinated and never-infected subjects, although effector T cell reactivity to the Omicron spike protein was higher than to the Wuhan Hu-1 spike protein (55), as in our experimental setting. A recent study suggested the existence of a unique insertion mutation in the Omicron spike protein that has a sequence that is identical to that of a coronavirus that causes the common cold (65), which may explain why T cells developed against common cold coronaviruses can cross-react with the SARS-CoV-2 spike protein (66–73). In light of these data and our present finding – of increased B and T cross-reactivity against Omicron in pre-pandemic samples from healthy donors – it is tempting to speculate that Omicron is acquiring mutations in the spike protein that are reminiscent, at least in part, of common cold coronaviruses, possibly explaining its increased infectivity but lower intrinsic virulence (74). At the time of writing, no study has addressed this issue and this topic is worth investigating further.

## Discussion

Collectively, all these data converge to demonstrate that primary mRNA vaccination is a potent tool for inducing long-lasting protection against severe disease outcomes – as was recently shown in a clinical setting (75, 76), particularly for previously infected subjects (77, 78). The booster, on the other hand, may provide additional protection in infection-naïve subjects. However, when it comes to highly contagious variants, such as Omicron and its subvariant (79), the risk of breakthrough infections remains high even following a booster-induced upsurge of neutralizing IgG antibodies (80). Indeed, all the available data suggest that vaccine-induced protection against infection is limited to 4/6 months, although protection against severe COVID-19 and death remained high (81). However, additional booster doses in high-risk subjects, such as the elderly and immunocompromised patients, are required to maintain protection against mortality associated with highly infectious SARS-CoV-2 variants (82, 83). However, in the general healthy population, future vaccination strategies should focus on identifying tools for achieving sterilizing immunity, including those that stimulate mucosal immunity (84–86), in order to avoid the need for repeated booster administration to keep antibody levels high and prevent infection. Needless to say, the development of a universal vaccine against all coronavirus strains could be an additional tool for preventing the spread of highly contagious future variants. The mosaic RBD nanoparticle vaccine has been shown in animal models to protect against challenges from diverse coronaviruses (87).

Limitations of the study: due to the observational, prospective nature of this cohort study, the following caveats must be considered. No evidence of a temporal relationship between exposure and outcome could be provided, as exposure and outcome were assessed simultaneously. The sample size was limited by expediency, although it is completely in line with all of the most recent studies in the field designed for deep immunological analysis of vaccinated individuals. Additionally, we enrolled all available HCWs who were offered the vaccination at the start of the vaccination campaign in Italy. Our study population may therefore be affected by selection bias, which limits how far the results can be extended to the general population, including different age groups. In our study, previously infected subjects had mainly been infected with Wuhan Hu-1, preventing us from identifying how different viral variants encountered during natural infection may shape vaccine responses, with possible implications for future next-generation vaccines (24, 88–91).

## Data availability statement

The original contributions presented in the study are included in the article/**Supplementary Material**. Further inquiries can be directed to the corresponding author.

## Ethics statement

The studies involving human participants were reviewed and approved by Ethical Committee of the Istituto Nazionale per le Malattie Infettive Lazzaro Spallanzani IRCCS (PARERE N. 444_2021). The patients/participants provided their written informed consent to participate in this study.

## Author contributions

Conceptualization: LP, FC, AB, GR. Methodology: LP, MT, FC, MM, AP, TP, ST, PT. Investigation: LP, MT, FC, MM, AP, TP, ST, PT. Supervision: AB, GR. Writing – original draft: LP, FC. Writing – review & editing: AB, GR. All authors contributed to the article and approved the submitted version.
