# Supplementary material for: Long-term adaptive response in COVID-19 vaccine recipients and the effect of a booster dose
Source: Front Immunol. 2023 Feb 28;14:1123158. doi: 10.3389/fimmu.2023.1123158 (PMC10011096; doi:10.3389/fimmu.2023.1123158)
Supplement: Supplementary file 1 [file Table_1.docx]

**Table S1.** Most commonly self-reported symptoms at the time of SARS-CoV-2 infection in previously infected subjects.

| Symptoms (%) | Previously infected  (n=17) |
| --- | --- |
| Required assistance from  the general practitioner (%) | 6 ( 35.3 ) |
| Required hospitalization (%) | 0 |
| Fever | 12( 70.6 ) |
| Shivering | 7 ( 41.2 ) |
| Headache | 9 ( 52.9 ) |
| Shortness of breath | 3 ( 17.6 ) |
| Chest pain | 1 ( 5.9 ) |
| Dry cough | 6 ( 35.3 ) |
| Productive cough | 2 ( 11.8 ) |
| Sore throat | 1 ( 5.9 ) |
| Cold | 2 ( 11.8 ) |
| Rhinorrhea | 3 ( 17.6 ) |
| Anosmia | 7 ( 41.2 ) |
| Ageusia | 5 ( 29.4 ) |
| Conjunctivitis | 0 |
| Muscular pain | 9 ( 52.9 ) |
| Fatigue | 10 ( 58.8 ) |
| Diarrhea | 2 ( 11.8) |
| Nausea | 0 |
| Vomiting | 0 |
| Lack of appetite | 5 ( 29.4) |
| Other | 2 ( 11.8 ) |
| No symptoms | 1 ( 5.9 ) |

**Table S2.** Correlation of anti-RBD neutralizing antibodies with RBD-specific memory B cells at T3

| **Group** | **variable (X)** | **variable (Y)** | ***p-value*** | **Pearson's correlation coefficient** |
| --- | --- | --- | --- | --- |
| Vaccinated subjects | anti-RBD antibodies | IgG^+^CD27^+^ RBD-B cells | 0.003 | 0.597 |
| Vaccinated subjects | anti-RBD antibodies | IgA^+^CD27^+^ RBD-B cells | 0.044 | 0.424 |
| Previously infected subjects | anti-RBD antibodies | IgG^+^CD27^+^ Omicron RBD-B cells | <0.001 | 0.837 |
